# On the Flow of a Cement Suspension: The Effects of Nano-Silica and Fly Ash Particles

**DOI:** 10.3390/ma17071504

**Published:** 2024-03-26

**Authors:** Chengcheng Tao, Mehrdad Massoudi

**Affiliations:** 1 School of Construction Management Technology, Purdue University, West Lafayette, IN 47907, USA; tao133@purdue.edu; 2 National Energy Technology Laboratory (NETL), U. S. Department of Energy, Pittsburgh, PA 15236, USA

**Keywords:** cement, rheology, nano-silica, fly ash, non-Newtonian fluids

## Abstract

Additives such as nano-silica and fly ash are widely used in cement and concrete materials to improve the rheology of fresh cement and concrete and the performance of hardened materials and increase the sustainability of the cement and concrete industry by reducing the usage of Portland cement. Therefore, it is important to study the effect of these additives on the rheological behavior of fresh cement. In this paper, we study the pulsating Poiseuille flow of fresh cement in a horizontal pipe by considering two different additives and when they are combined (nano-silica, fly ash, combined nano-silica, and fly ash). To model the fresh cement suspension, we used a modified form of the power-law model to demonstrate the dependency of the cement viscosity on the shear rate and volume fraction of cement and the additive particles. The convection–diffusion equation was used to solve for the volume fraction. After solving the equations in the dimensionless forms, we conducted a parametric study to analyze the effects of nano-silica, fly ash, and combined nano-silica and fly ash additives on the velocity and volume fraction profiles of the cement suspension. According to the parametric study presented here, larger nano-silica content results in lower centerline velocity of the cement suspension and larger non-uniformity of the volume fraction. Compared to nano-silica, fly ash exhibits an opposite effect on the velocity. Larger fly ash content results in higher centerline velocity, while the effect of the fly ash on the volume fraction is not obvious. For cement suspension containing combined nano-silica and fly ash additives, nano-silica plays a dominant role in the flow behavior of the suspension. The findings of the study can help the design and operation of the pulsating flow of fresh cement mortars and concrete in the 3D printing industry.

## 1. Introduction

There is a strong demand to improve the rheological properties of cement and concrete materials including ultra-high performance concrete, high-strength concrete, self-consolidated concrete, and concrete for 3D printing in the industry. The cement and concrete design with improved rheology is important to enhance constructability and workability, facilitate processing and mixing, and enhance in situ material performance [1]. The addition of nano-additives, such as nano-silica, graphene oxide, nano-TiO_2_, and cellulose nanofibers (CNFs) can significantly modify and improve the rheological properties of cement-based materials; these additives can also affect the yield stress, the plastic viscosity, the rate of structural buildup at rest, the degree of hydration, mechanical properties, and durability [2,3,4,5,6,7]. Various factors including the nanomaterial type, constituent, degree of dispersion, mixing energy, high-range water reducer, sonication, and water-to-cement ratio (*w*/*c*) can affect the rheology of fresh cement and concrete. The combined effects of these various factors would be quite complex. Among different nanoparticles used in cement and concrete materials, nano-silica or nano-SiO_2_ is the most commonly used nano-additive for performance improvement, mainly due to the pozzolanic reactivity and the pore-filling effect [8]. According to Lavergne, et al. [9], nano-silica particles accelerated the hydration of the cement paste and increased the shear strength and early strength significantly because of the specific surface of the nano-silica. Kim, et al. [10] indicated that the addition of nano-silica and silica fume to the high-volume fly ash cement (HVFC) accelerated the initial hydration reaction of HVPC, resulting in a shorter setting time, and higher compressive strength during the initial and middle stages of hydration. The high viscosity of alkali silicate-activated cements has been a big issue that prevents the wide application of this type of material [11]. Fly ash additives have been widely used in cement, concrete, and asphalt industries to improve rheology and workability by decreasing yield stress and viscosity [12,13,14,15,16,17,18]. Fly ash microspheres (FAMs) with highly spherical particles work as ball bearings in fresh cement suspensions by reducing the internal friction between the additives, mitigating the floc agglomeration, and reducing locked water [11]. Wang, et al. [13] concluded that the addition of fly ash increased the fluidity of graphene oxide-cement paste and improved the rheological properties of the cement paste with fly ash and graphene oxide additives. Fly ash plays an economical and effective role in achieving the intended properties of graphene oxide-cement paste because of its ball effect, size gradation, and less water requirement. Golewski [19] illustrated the combined effect of different additives, including siliceous fly ash, silica fume, and nano-silica, which accelerated the hardening of concrete after 28 days of curing and reduced the microcracks in the interfacial transition zone between the aggregates and the cement paste. García-Taengua, et al. [20] investigated the effect of nano-silica and fly ash on the rheology of fresh cement mortars. The study showed that the viscosity reached its maximum at 2% nano-silica content, and larger fly ash content increased the viscosity when used together with the nano-silica additives. In addition, researchers have used various additives such as viscosity modifying agents, superplasticizers, and organic chemical admixtures [21,22,23,24]. In this paper, we are focusing on nano-additives and fly ashes since they have been widely used in cementitious materials and exhibit effectiveness in improving the rheological properties of fresh cement and concrete.

Computational modeling has been considered a useful tool to simulate the behavior of cement and concrete materials [25,26]. Rheological models can provide some necessary information for the fluid behavior of fresh cement slurries [27]. In general, fresh cement slurries are considered to behave as non-Newtonian fluids [28]. In our previous studies [29,30,31], we reviewed the rheological models for fresh cement by considering various effects on viscosity and yield stress, and conducted parametric studies for steady and pulsating Poiseuille flow of fresh cement modeled as non-Newtonian fluids. The study of the pulsating flow of fresh cement materials provides useful information for the 3D-printed cement and concrete industry by considering the vibration/oscillatory effect. Researchers have conducted experimental investigations and summarized the effect of different additives on the viscosity and yield stress of fresh cementitious suspensions. However, most of the existing models of cement suspensions with additives are obtained from experimental data in one-dimensional form. There is a lack of a comprehensive rheological model with three-dimensional (3D) tensor notation where the non-Newtonian nature of the cement suspensions with additives is considered. In addition, there is no constitutive relation that considers the effect and the significance of shear rate, volume fraction, and additives such as nano-silica, fly ashes, and combined additives on the flow of fresh cement suspensions.

In this paper, we look at the pulsating flow of a cement suspension in a pipe with two additives and when they are combined: (1) nano-silica, (2) fly ash, and (3) nano-silica and fly ash. The fresh cement is modeled as a non-Newtonian fluid. We use a modified form of the power-law model to study the dependency of the cement suspension viscosity on the shear rate and volume fraction of the cement and additive particles. The volume fraction of particles is solved through a convection–diffusion equation. In Section 2, the governing equations of motion are presented, assuming no thermal or chemical effects (isothermal conditions). In Section 3, we briefly discuss the constitutive equations for the viscous stress tensor and the diffusive flux vector. In Section 4, we solve for the dimensionless form of the governing equations and provide numerical solutions in the dimensionless form. A limited parametric study is performed for a limited range of different dimensionless numbers. In Section 5, we present a few concluding remarks.

## 2. Governing Equations

A fresh cement suspension, in general, behaves like a multiphase fluid composed of different materials such as cement particles, water, additives, etc. To model it mathematically, there are many approaches; for example, the multicomponent (Mixture Theory) approach can be used, where there is a two-way coupling between the two phases (constituents). In this approach, the governing equations are written for each component. Consequently, constitutive equations are needed for the stress tensors, along with the interaction forces (see [32,33]). Alternatively, to simplify things, the cement suspension can be assumed to behave like a non-homogeneous fluid suspension with microstructure because of the presence of the cement particles and other added particles. In this approach, the material parameters, such as viscosity, may depend on the particle concentration, also known as the volume fraction of the particles. This approach has been advocated by Tao et al. [34] and we will use the same approach here. For an isothermal case, the governing equations are the balance equations for mass, linear momentum, angular momentum, and the convection–diffusion for the particle flux [35].

### 2.1. Conservation of Mass

(1) $$\frac{\partial \rho }{\partial t}+\mathrm{div}\left(\rho v\right)=0$$

where ∂/∂t is the partial derivative with respect to time, div is the divergence operator, ρ is the density of the fresh cement suspension, which is related to the density of water and the cement particles, and v is the velocity vector. Notice that now we have a new parameter, namely the volume fraction ϕ of the (cement) particles. This function has the property that $0\leq \phi (x,t)\leq \phi_{max}<1$; that is, its value is either one or zero. Since ϕ(x,t) is a smooth function, we, in a sense, homogenize the solid particle distribution; thus, the shape and size of the particles do not have any direct impact on the constitutive formulation. As a result, the density of the suspension is given by $\rho =\rho_{f0}\left(1-\phi \right)+\rho_{s0}\phi$, where $\rho_{f0}$ and $\rho_{s0}$ are the pure density of water and the cement particles before mixing with water, respectively. The fresh cement suspension is assumed to behave as an incompressible fluid, that is

(2) div v=0

### 2.2. Conservation of Linear Momentum

(3) $$\rho \frac{dv}{dt}=\mathrm{div}T+\rho b$$

where d/dt is the total time derivative, given by $\frac{d\left(.\right)}{dt}=\frac{\partial (.)}{\partial t}+\left[\mathrm{grad}\left(.\right)\right]v$, grad is the gradient operator, b represents the body force, and T is the stress tensor.

### 2.3. Conservation of Angular Momentum

The angular momentum equation indicates that the stress tensor is symmetric if there are no couple stresses, namely

(4) $$T=T^{T}$$

### 2.4. Convection–Diffusion Equation

For the motion of the particles, a convection–diffusion equation is used; the particles move with the velocity of the suspension (see [36,37]).

(5) $$\frac{\partial \phi }{\partial t}+\mathrm{div}(\phi v)=-divN$$

where ∂ϕ/∂t is the rate of accumulation of particles, div(ϕv) represents particle motion due to the bulk flow, and divN is the diffusive flux.

In addition to the governing equations above, we also need constitutive relations for T and N. In the next section, we discuss the constitutive relations of the stress tensor T and the flux vector N.

## 3. Constitutive Relations

### 3.1. Stress Tensor

A cement suspension generally behaves as a complex fluid that has a yield stress, and oftentimes exhibits thixotropy, viscoelasticity, etc. For cement suspensions, the formulation of the stress tensor significantly affects the properties and the workability of fresh and hardened materials. It is usually assumed that the stress tensor has two parts: $T=T_{y}+T_{v}$, with $T_{y}$ being the yield stress and $T_{v}$ the viscous stress. Tao et al. [29] gave a comprehensive review of the constitutive relations for fresh cement suspensions. Tao et al. [34], based on the model proposed by Massoudi and Phuoc [38], suggested a general constitutive relation [39,40]. In this paper, a simplified version of this model for the viscous stress $T_{v}$ is used,

(6) $$T_{v}=-pI+\mu_{\mathrm{eff}}\left(\phi ,A_{1}\right)A_{1}$$

where p is the pressure (the mean value of the stress tensor), I is the identity tensor, and $\mu_{\mathrm{eff}}$ represents the effective shear viscosity (a function of the volume fraction ϕ and the shear rate). The tensor $A_{1}$ is given as

(7) $$A_{1}=\mathrm{grad}v+{\left(\mathrm{grad}v\right)}^{T}$$

Bridges and Rajagopal [41] used an effective viscosity relationship:

(8) $$\mu_{\mathrm{eff}}\left(\phi ,A_{1}\right)=\mu^{*}(\phi ){\left[1+\alpha tr{A_{1}}^{2}\right]}^{m}$$

where $\mu^{*}\left(\phi \right)$ is assumed to be given by the Batchelor and Green [42]:

(9) $$\mu^{*}\left(\phi \right)=\mu_{0}\left(1+2.5\phi +7.6\phi^{2}\right)$$

where $\mu_{0}$ is the viscosity of the base fluid [43]. Equation (8) follows the traditional power-law model, where the viscosity depends on the shear rate. When *m* < 0, the suspension behaves as a shear-thinning fluid, and when *m* > 0, it behaves as a shear-thickening one; and α is related to the consistency index. There are other correlations for $\mu^{*}\left(\phi \right)$ (see Krieger and Dougherty’s model [44]).

By substituting Equations (8) and (9) into (6), the viscous stress tensor is given:

(10) $$T_{v}=-pI+\mu_{0}\left(1+2.5\phi +7.6\phi^{2}\right){\left[1+\alpha tr{A_{1}}^{2}\right]}^{m}A_{1}$$

Equation (10) will be used as the starting point of our modeling. We will modify this equation by incorporating the effects of the nano-silica and the fly ash particles. In this paper, we ignore the thixotropic effect and the yield stress [28,45].

#### 3.1.1. Nano-SiO_2_ Additive

With the emergence of nanotechnology as a new and promising technology, nanomaterials have been introduced to improve the strength of cementitious materials. Nano-SiO_2_ (NS) particle is one of the most commonly used nanomaterials in cement. In this paper, we use a viscosity relationship that considers the effect of a hydrophilic NS [46]. The mean diameter of the NS is 12 nm, with a specific surface area of 200 m^2^/g, density of 2.2 g/cm^3^, pH of 4.5, with a purity greater than 99.8%. The cement suspension used in the model has the following composition: CaO–65.0%, SiO_2_–21.2%, Al_2_O_3_–5.16%, Fe_2_O_3_–3.39%, SO_3_–2.43%, MgO–1.32%, K_2_O–0.63%, TiO_2_–0.78%, and Na_2_O–0.07%.

Equation (10) is now modified to include the effect of NS particles, obtaining the equation suggested by Ouyang et al. [46]. This viscous stress tensor is shown in Equation (11),

(11) $$T_{v}=-pI+\mu_{0}\left(\frac{1}{1-2.5{{\left(1+\frac{r_{ns}}{R_{ns}}\right)}^{3}\phi }_{NS/W}}\right)(1+2.5\phi +7.6\phi^{2}){\left[1+\alpha tr{A_{1}}^{2}\right]}^{m}A_{1}$$

where, $r_{ns}$ is the adsorbed water layer thickness for the nanoparticle, $R_{ns}$ is the diameter of nanoparticles, $\phi_{NS/W}$ is the ratio of nano-silica particle volume to the total volume of nano-silica suspension.

#### 3.1.2. Fly Ash Additive

Fly ash (FA) is another commonly used additive in cement; it is primarily used to enhance rheology and the workability of cement. Based on the fly ash study by Sybertz and Reick [18], the viscous stress tensor as given in Equation (10) is modified to include the effect of fly ash additive, as shown in Equation (12). The fly ash we consider in this paper has the following properties: density of Fly Ash I–2320 kg/m^3^, density of Fly Ash II–2380 kg/m^3^, surface area from Blaine analysis of Fly Ash I is 309 m^2^/kg, and surface area from Blaine analysis of Fly Ash II is 332 m^2^/kg. The cement suspension model now has the following form:

(12) $$T_{v}=-pI+\mu_{0}(Q_{1}e^{Q_{2}\phi }\left(1-\phi_{f}\right)+Q_{3}e^{Q_{4}\phi }\phi_{f}){(1+2.5\phi +7.6\phi^{2})\left[1+\alpha tr{A_{1}}^{2}\right]}^{m}A_{1}$$

where, $\phi_{f}$ is the fly ash content and $Q_{n}$ (n = 1, 2, 3, 4) are the parameters from experimental fitting, where $Q_{1}$ = 2.31 $\times \;10^{-3}$ (Fly Ash I) and 4.75 $\times \;10^{-3}$ (Fly Ash II), $Q_{2}$ = 20.7 (Fly Ash I) and 19.1 (Fly Ash II), $Q_{3}$ = 7.96 $\times \;10^{-5}$ (Fly Ash I) and 1.29 $\times \;10^{-5}$ (Fly Ash II), and $Q_{4}$ = 17 (Fly Ash I) and 18.9 (Fly Ash II) [18].

#### 3.1.3. Combined Nano-SiO_2_ and Fly Ash Additives

Considering that both the nano-SiO_2_ particles and fly ash particles are added to the cement suspension, the viscous stress tensor for the NSFA-added case is shown in Equation (13),

(13) $$\begin{array}{c} T_{v}=-pI+\mu_{0}\left(\frac{1}{1-2.5{{\left(1+\frac{r_{ns}}{R_{ns}}\right)}^{3}\phi }_{NS/W}}\right)(1+2.5\phi +7.6\phi^{2})((Q_{1}e^{Q_{2}\phi }\left(1-\phi_{f}\right)\\ +Q_{3}e^{Q_{4}\phi }\phi_{f}){\left[1+\alpha tr{A_{1}}^{2}\right]}^{m}A_{1} \end{array}$$

where the meaning of the parameters is as described previously. We use these three stress tensor equations in our analysis.

### 3.2. Particle Flux

In our earlier paper, we used a correlation based on the work of Philips et al. [47]. Here, we assume that N is given by [41]:

(14) N=−D∇ϕ

where D is the diffusion coefficient or the diffusivity, and ∇ is the gradient operator. We also assume that *D* has two parts: a shear-rate-dependent part and a concentration-dependent part:

(15) $$D=D\left(\dot{\gamma },\phi \right)=D_{1}(\dot{\gamma })D_{2}(\phi )$$

For $D_{1}(\dot{\gamma })$, we have [41]:

(16) $$D_{1}(\dot{\gamma })=\eta \left\|{A_{1}}^{2}\right\|$$

where η is a constant, and $\left\|\cdot \right\|$ is the trace-norm, where $\left\|{A_{1}}^{2}\right\|={\left(\frac{\partial v}{\partial r}\right)}^{2}$ for the one-dimensional flow of a suspension in a pipe. For $D_{2}(\phi )$, based on [48], we use the relation suggested by [42,49], where for a dense suspension:

(17) $$D_{2}\left(\phi \right)=D_{0}\left[{\left(1-\phi \right)}^{2.55}\left(1+4\phi +4\phi^{2}-4\phi^{3}+\phi^{4}\right)\right]$$

where $D_{0}$ is a constant. Substituting Equations (16) and (17) in Equation (14), we have the following:

(18) $$N=-D_{0}\eta \left\|{A_{1}}^{2}\right\|\left[{\left(1-\phi \right)}^{2.55}\left(1+4\phi +4\phi^{2}-4\phi^{3}+\phi^{4}\right)\right]\nabla \phi$$

We use Equation (18) in this paper.

## 4. Results and Discussion

**Figure 1:**
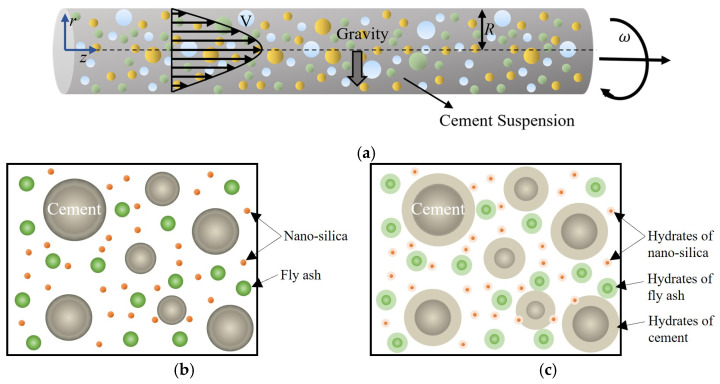
(**a**) Schematic of the horizontal pipe flow of cement suspension; (**b**) cement suspension with nano-silica and fly ash additives; (**c**) hydration of cement, nano-silica, and fly ash.

Here, we study the pulsating flow of a cement suspension in a horizontal pipe. Figure 1 shows the schematic of the horizontal pipe flow of cement suspension with nano-silica and fly ash additives.

### 4.1. Problem Statement

We assume that the velocity and the volume fraction fields can be expressed as follows:

(19) $$\left\{\begin{array}{c} \phi =\phi (r,t)\\ v=v\left(r,t\right)e_{z} \end{array}\right.$$

Indicating that an unsteady flow is in the *z*-direction, where the velocity and the volume fraction depend on time and the radius. With the assumptions above, Equation (1) is satisfied.

By substituting the constitutive Equations (11)–(13) into the governing Equation (3), the linear momentum equations are reduced to (using the cylindrical coordinate system)

(20) $$\frac{\partial p}{\partial r}+\rho g=0$$

(21) $$-\frac{1}{r}\frac{\partial p}{\partial \theta }=0$$

(22) $$\frac{1}{r}\frac{\partial }{\partial r}\left\{r\mu^{**}(\phi ){\left[1+2\alpha {\left(\frac{\partial v}{\partial r}\right)}^{2}\right]}^{m}\frac{\partial v}{\partial r}\right\}-\frac{\partial p}{\partial z}=\rho \frac{\partial v}{\partial t}$$

When NS particles are added to the cement suspension, $\mu^{**}\left(\phi \right)$ becomes

(23) $$\mu^{**}\left(\phi \right)=\mu_{0}\left(\frac{1}{1-2.5{{\left(1+\frac{r_{ns}}{R_{ns}}\right)}^{3}\phi }_{NS/W}}\right)\left(1+2.5\phi +7.6\phi^{2}\right)$$

When FA particles are added to the cement suspension, $\mu^{**}\left(\phi \right)$ becomes

(24) $$\mu^{**}\left(\phi \right)=\mu_{0}\left(Q_{1}e^{Q_{2}\phi }\left(1-\phi_{f}\right)+Q_{3}e^{Q_{4}\phi }\phi_{f}\right)\left(1+2.5\phi +7.6\phi^{2}\right)$$

And, when both NS and FA particles are added to the cement suspension, $\mu^{**}\left(\phi \right)$ becomes

(25) $$\mu^{**}\left(\phi \right)=\mu_{0}\left(\frac{1}{1-2.5{{\left(1+\frac{r_{ns}}{R_{ns}}\right)}^{3}\phi }_{NS/W}}\right)\left(Q_{1}e^{Q_{2}\phi }\left(1-\phi_{f}\right)+Q_{3}e^{Q_{4}\phi }\phi_{f}\right)(1+2.5\phi +7.6\phi^{2})$$

Equations (20) and (21) show the variation of the pressure in the *r*- and θ-directions, respectively. To look at the effects of the pressure, based on previous studies, it is assumed that the axial pressure gradient is expressed as follows [41]:

(26) $$\frac{\partial p}{\partial z}=-\left[A_{0}+B_{0}\sin\omega t\right]$$

where $A_{0}$ and $B_{0}$ are constants and ω is the pulsating frequency. By substituting Equation (26) into (22), we have

(27) $$\frac{1}{r}\frac{\partial }{\partial r}\left\{r\mu^{**}\left(\phi \right){\left[1+2\alpha {\left(\frac{\partial v}{\partial r}\right)}^{2}\right]}^{m}\frac{\partial v}{\partial r}\right\}+A_{0}+B_{0}\sin\omega t=\left[\rho_{f0}\left(1-\phi \right)+\rho_{s0}\phi \right]\frac{\partial v}{\partial t}$$

By substituting Equation (15) into the convection–diffusion Equation (5), we obtain

(28) $$\frac{1}{r}\frac{\partial }{\partial r}\left\{r\cdot \eta \cdot {\left(\frac{\partial v}{\partial r}\right)}^{2}\cdot D_{0}\left[{\left(1-\phi \right)}^{2.55}\left(1+4\phi +4\phi^{2}-4\phi^{3}+\phi^{4}\right)\right]\frac{\partial \phi }{\partial r}\right\}=\frac{\partial \phi }{\partial t}$$

The above-coupled equations need to be solved numerically. Before solving Equations (27) and (28), we will obtain their dimensionless forms by non-dimensionalizing the equations through:

(29) $$\bar{r}=\frac{r}{R}\;\bar{v}=\frac{v}{V}\;\bar{t}=\frac{Vt}{R}\;\bar{\omega }=\frac{R\omega }{V}$$

where *R* is a reference length (the radius) and *V* is a reference velocity. The dimensionless forms of Equations (27) and (28) become

(30) $$\begin{array}{ll} \left[\left(1-\phi \right)+\frac{\rho_{s0}}{\rho_{f0}}\phi \right] & \frac{\partial \bar{v}}{\partial \bar{t}}\\ & =\frac{1}{Re}\frac{1}{\bar{r}}\frac{\partial }{\partial \bar{r}}\left\{\bar{r}X_{add}\left(1+2.5\phi +7.6\phi^{2}\right){\left[1+\delta {\left(\frac{\partial \bar{v}}{\partial \bar{r}}\right)}^{2}\right]}^{m}\frac{\partial \bar{v}}{\partial \bar{r}}\right\}\\ & +P\left(1+\zeta \sin\bar{\omega }\bar{t}\;\right) \end{array}$$

(31) $$\frac{\partial \phi }{\partial \bar{t}}=\frac{\kappa }{\bar{r}}\frac{\partial }{\partial \bar{r}}\left\{\bar{r}\cdot \left[{\left(1-\phi \right)}^{2.55}\left(1+4\phi +4\phi^{2}-4\phi^{3}+\phi^{4}\right)\right]{\left(\frac{\partial \bar{v}}{\partial \bar{r}}\right)}^{2}\frac{\partial \phi }{\partial \bar{r}}\right\}$$

where

(32) $$Re=\frac{\rho_{f0}VR}{\mu_{0}};\delta =2\alpha \frac{V^{2}}{R^{2}};P=\frac{A_{0}R}{\rho_{f0}V^{2}};G_{0}=\frac{gR}{V^{2}};\kappa =\frac{\eta D_{0}}{RV};\;\zeta =\frac{B_{0}}{A_{0}};\;\chi =\frac{\rho_{s0}}{\rho_{f0}}$$

where Re is the Reynolds number, the ratio of the forces due to inertia to the viscous effects, δ is related to the shear rate, *P* to the pulsation pressure, $G_{0}$ is related to the gravity term, κ to the cement suspension diffusivity and the volume fraction, ζ is related to the terms in the pulsating pressure, and χ represents the ratio of the two densities.

In Equation (30), when NS particles are added to the cement suspension, $X_{add}=\left(\frac{1}{1-2.5{{\left(1+\frac{r_{ns}}{R_{ns}}\right)}^{3}\phi }_{NS/W}}\right)$. And, when FA particles are added to the cement suspension, $X_{add}=\left(Q_{1}e^{Q_{2}\phi }\left(1-\phi_{f}\right)+Q_{3}e^{Q_{4}\phi }\phi_{f}\right)$. When NS and FA particles are added to the cement suspension, $X_{add}=\left(\frac{1}{1-2.5{{\left(1+\frac{r_{ns}}{R_{ns}}\right)}^{3}\phi }_{NS/W}}\right)\left(Q_{1}e^{Q_{2}\phi }\left(1-\phi_{f}\right)+Q_{3}e^{Q_{4}\phi }\phi_{f}\right)$.

From the above equations, it is obvious that two boundary conditions are needed for the velocity and two for the volume fraction. Initial conditions also need to be specified for v and ϕ.

### 4.2. Boundary and Initial Conditions

We impose the no-slip boundary condition and we use the symmetry condition as indicated in Equation (33).

(33) $$\bar{v}\left(1,\bar{t}\right)=0\;\frac{\partial \phi }{\partial \bar{r}}\left(1,\bar{t}\right)=0$$

For the volume fraction ϕ, an average value is assumed, $\phi_{avg}$ [34], where

(34) $$\int_{0}^{1}\phi d\bar{r}=\phi_{avg}$$

For the initial conditions, based on [41], we apply a uniform value for the velocity at *t* = 0 and impose a parabolic profile for the volume fraction [41].

(35) $$\bar{v}\left(\bar{r},0\right)=1\;\phi \left(\bar{r},0\right)={\bar{r}}^{2}\left(3-2\bar{r}\right)$$

According to Equation (35), this initial concentration near the centerline is smaller than the concentration of the particles at the wall. We carried out some tests for a uniform volume fraction profile at *t* = 0 and we did not notice any noticeable deviation from the suggested equation given in Equation (35).

Also, the flow rate or the flow output is defined as,

(36) $$\bar{Q}(\bar{t})=2\pi \int_{0}^{1}\bar{v}\left(\bar{r},\bar{t}\right)\bar{r}d\bar{r}$$

### 4.3. Numerical Results and Discussion

MATLAB (R2022a) *pdepe* is a function that is used to solve initial-boundary value problems for time-dependent partial differential equations (PDEs) that describe various physical phenomena. This solver is particularly useful for problems with one spatial dimension and time evolution. The above dimensionless partial differential equations are solved using the MATLAB *pdepe* solver for one spatial variable $\bar{r}$ and time $\bar{t}$. The step size is the default value used in the solver. In this section, we will present the results for the velocity and volume fraction profiles for a limited range of dimensionless numbers. In this paper, we focus on the effects of $\phi_{NS/W}$ (the ratio of nano-silica particle volume to the total volume of nano-silica) and $\phi_{f}$ (the fly ash content); by varying these parameters, we look at the effects on the velocity and the volume fraction fields. In this section, a parametric study is performed to investigate the effect of different volume fractions of the additives at different time cycles on the behavior of fresh cement suspension. Table 1 shows the values of the volume fraction of the additives and the dimensionless parameters.

#### 4.3.1. NS-Added Particles to the Cement Suspension

**Figure 2:**
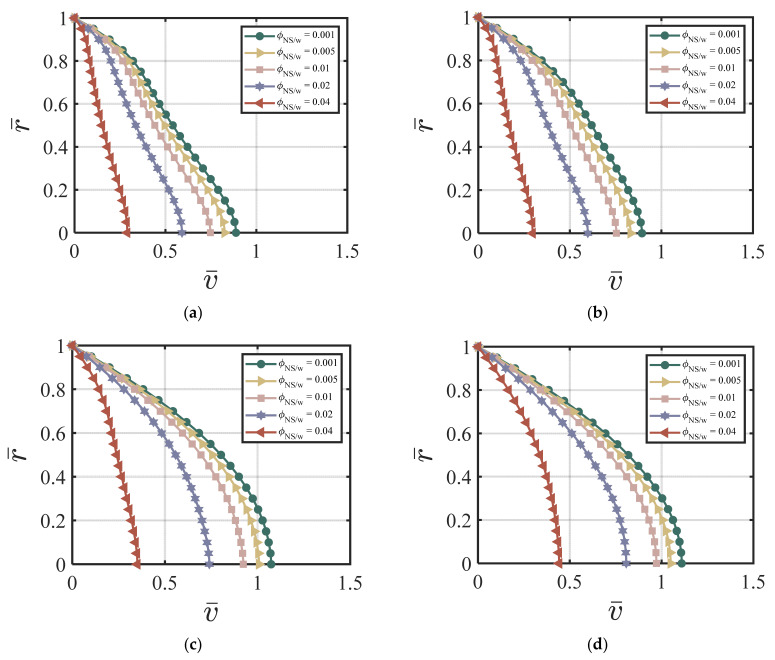
Effect of $\phi_{NS/W}$ on the velocity at (**a**) cycle #2; (**b**) cycle #10; (**c**) cycle #50; (**d**) cycle #220 with $\phi_{avg}=0.4$, k = 6.5, *χ* = 3.15, *ζ* = 1, m = 0.5, *Re* = 100, *P* = 10, $G_{0}$ = 0, *κ* = 0.001.

**Figure 3:**
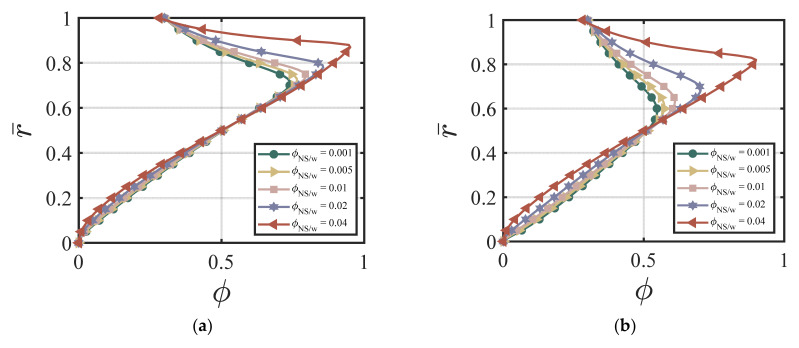

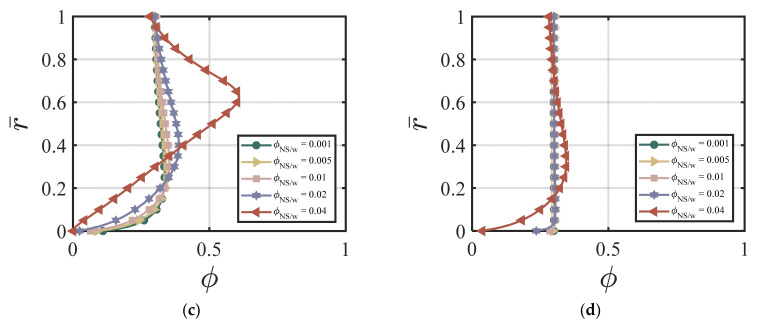
Effect of $\phi_{NS/W}$ on the volume fraction at (**a**) cycle #2; (**b**) cycle #10; (**c**) cycle #50; (**d**) cycle #220 with $\phi_{avg}=0.4$, k = 6.5, *χ* = 3.15, *ζ* = 1, m = 0.5, *Re* = 100, *P* = 10, $G_{0}$ = 0, *κ* = 0.001.

**Figure 4:**
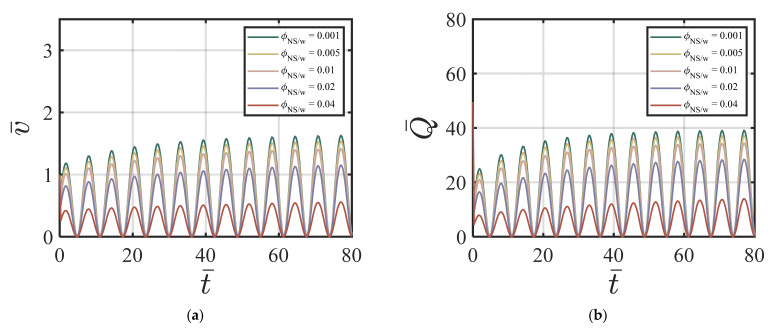
Effect of $\phi_{NS/W}$ on (**a**) $\bar{v}$ vs. *t*; (**b**) $\bar{Q}$ vs. *t* with $\phi_{avg}=0.4$, k = 6.5, *χ* = 3.15, *ζ* = 1, m = 0.5, *Re* = 100, *P* = 10, $G_{0}$ = 0, *κ* = 0.001.

In this section, we demonstrate the effect of nano-silica content on the velocity and the cement volume fraction. As an example, we chose the following dimensionless values for our numerical simulations: $\phi_{avy}=0.4$, k = 6.5, *χ* = 3.15, *ζ* = 1, m = 0.5, *Re* = 100, *P* = 10, $G_{0}$ = 0, *κ* = 0.001. Figure 2a–d show the effects of different $\phi_{NS/W}$ on the velocity at four different time cycles (cycle #2, #10, #50, and #220). According to Figure 2a–d, larger $\phi_{NS/W}$ values produce a lower centerline velocity. The velocity profile reaches a steady state and does not change much after 50 time cycles. Figure 3a–d show the effects of different $\phi_{NS/W}$ on the cement volume fraction at four different time cycles (cycle #2, #10, #50, and #220). The results show that higher values of $\phi_{NS/W}$ results in larger non-uniformity of the volume fraction, especially at the early stages. The volume fraction profiles reach a steady state after 220 time cycles. Figure 4a,b show the effects of $\phi_{NS/W}$ on the velocity versus time cycle and the flow rate versus time cycle. The fresh cement suspension with higher nano-silica content reaches the steady state condition faster than those with lower nano-silica content. The results align well with the findings in the literature [9,50,51,52,53] for the effect of nano-silica additives on the early-stage performance of fresh cement suspension. According to Lavergne, et al. [9] and Kim, et al. [10], nano-silica particles accelerated the initial hydration reaction of the cement suspension and increased the shear strength and the early strength significantly because of the specific surface of the nanoparticles, leading to a shorter time of set and a higher compressive early strength.

#### 4.3.2. FA-Added Particles to the Cement Suspension

**Figure 5:**
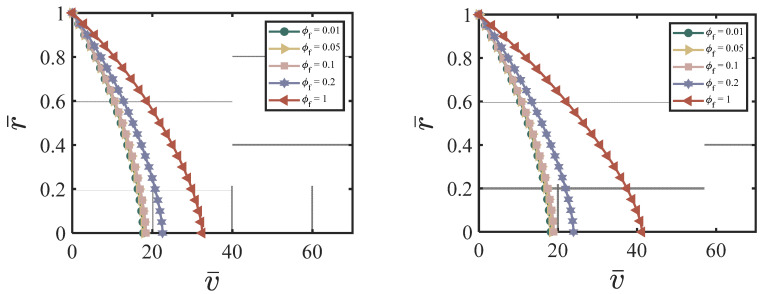

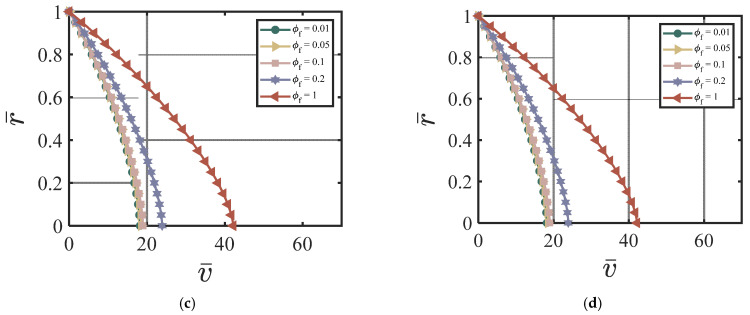
Effect of $\phi_{f}$ on the velocity at (**a**) cycle #5; (**b**) cycle #10; (**c**) cycle #50; (**d**) cycle #200 with $\phi_{avg}=0.4$, $Q_{1}=4.04\times 10^{-5}$, $Q_{2}=19$, $Q_{3}=0.54\times 10^{-5}$, $Q_{4}=20.9$, *χ* = 3.15, *ζ* = 1, m = 0.5, *Re* = 100, *P* = 10, $G_{0}$ = 0, *κ* = 0.001.

**Figure 6:**
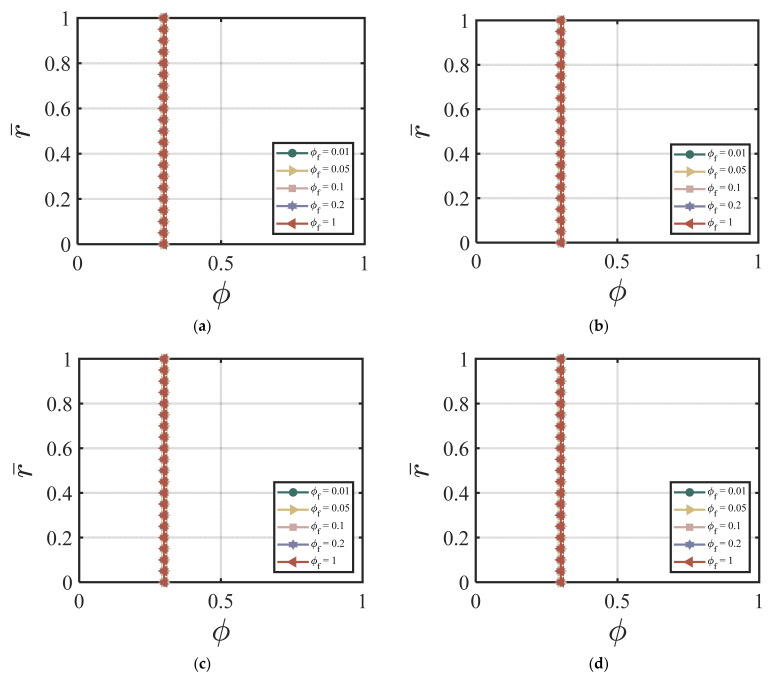
Effect of $\phi_{f}$ on the volume fraction at (**a**) cycle #5; (**b**) cycle #10; (**c**) cycle #50; (**d**) cycle #200 with $\phi_{avg}=0.4$, $Q_{1}=4.04\times 10^{-5}$, $Q_{2}=19$, $Q_{3}=0.54\times 10^{-5}$, $Q_{4}=20.9$, *χ* = 3.15, *ζ* = 1, m = 0.5, *Re* = 100, *P* = 10, $G_{0}$ = 0, *κ* = 0.001.

**Figure 7:**
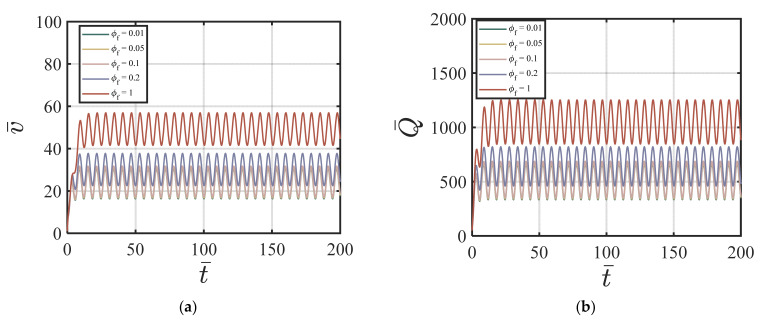
Effect of $\phi_{f}$ on (**a**) $\bar{v}$ vs. *t*; (**b**) $\bar{Q}$ vs. *t* with $\phi_{avg}=0.4$, $Q_{1}=4.04\times 10^{-5}$, $Q_{2}=19$, $Q_{3}=0.54\times 10^{-5}$, $Q_{4}=20.9$, *χ* = 3.15, *ζ* = 1, m = 0.5, *Re* = 100, *P* = 10, $G_{0}$ = 0, *κ* = 0.001.

Here, we use the following values for our numerical simulations: $\phi_{avy}=0.4$, $Q_{1}=4.04\times 10^{-5}$, $Q_{2}=19$, $Q_{3}=0.54\times 10^{-5}$, $Q_{4}=20.9$, *χ* = 3.15, *ζ* = 1, m = 0.5, *Re* = 100, *P* = 10, $G_{0}$ = 0, *κ* = 0.001. Figure 5a–d show the effects of different values of $\phi_{f}$ on the velocity and the volume fraction at four different time cycles #5, #10, #50, and #200. According to Figure 5a–d, a higher $\phi_{f}$ value causes a higher centerline velocity, which exhibits an opposite trend compared to the nano-silica additives in Figure 2a–d. The velocity profile reaches a steady state after 20 time cycles. Figure 6a–d show the effects of different values of $\phi_{f}$ on the volume fraction at four different time cycle #5, #10, #50, and #200. The effect of the fly ash particles on the volume fraction is not that obvious. And, the volume fraction field reaches its steady state value very fast (within 5 time cycles). Figure 7a,b show the effects of $\phi_{f}$ on the velocity versus time cycle and the flow rate versus time cycle. In contrast to the cement suspension with nano-silica additives, the cement suspension with lower fly ash content reaches the steady state condition faster than those with higher fly ash content. The behavior agrees with the findings by Wang, et al. [50], who indicated that the effect of nano-silica on cement hydration and performance happened in the early stages while high content of fly ash contributed to the strength growth in the later stages. The cement suspension with higher nano-silica content reaches the steady state condition faster than those with lower nano-silica content. In addition, the findings align well with the study by García-Taengua, et al. [20], who indicated that fly ash affected the viscosity only when used together with nano-silica additives, and the fly ash did not affect the dormant period of cement hydration.

#### 4.3.3. Combined NS and FA-Added Particles to the Cement Suspension

**Figure 8:**
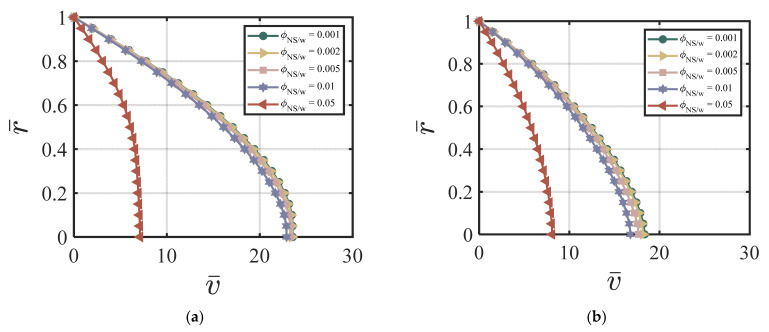

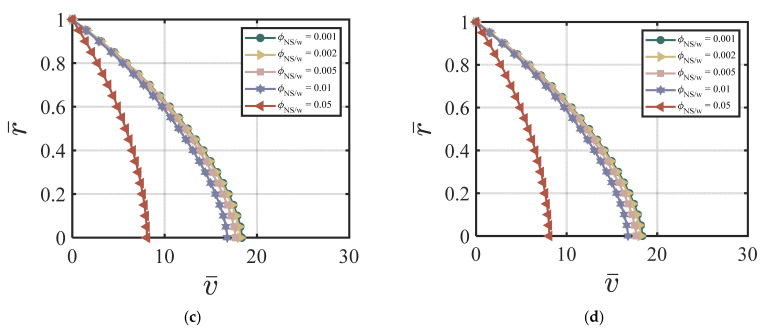
Effect of $\phi_{NS/W}$ of the combined NS FA-added cement suspension on velocity at (**a**) cycle #2; (**b**) cycle #10; (**c**) cycle #50; (**d**) cycle #200 with $\phi_{avy}=0.4$, k = 6.5, $Q_{1}=4.04\times 10^{-5}$, $Q_{2}=19$, $Q_{3}=0.54\times 10^{-5}$, $Q_{4}=20.9$, *χ* = 3.15, *ζ* = 1, m = 0.5, *Re* = 100, *P* = 10, $G_{0}$ = 0, *κ* = 0.001.

**Figure 9:**
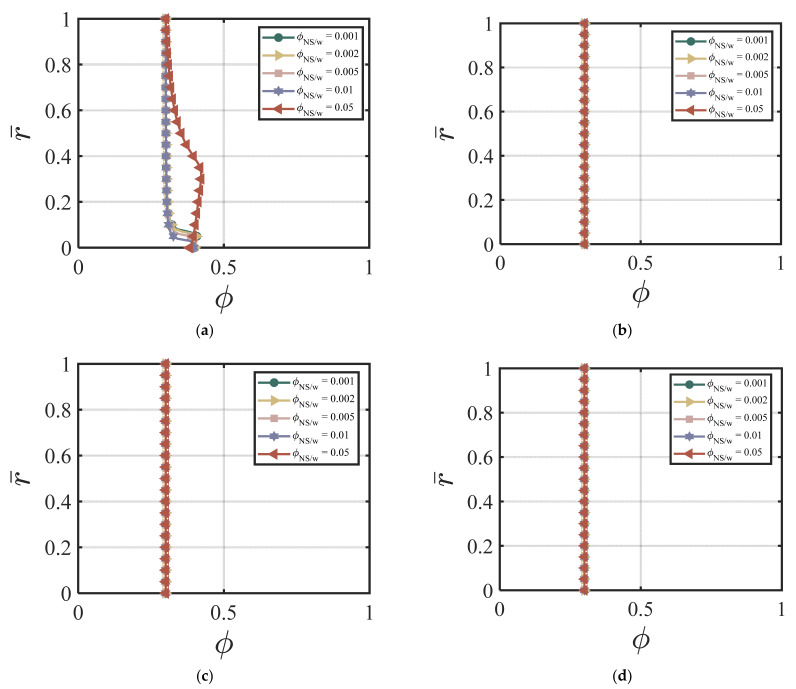
Effect of $\phi_{NS/W}$ of the combined NS FA-added cement suspension on volume fraction at (**a**) cycle #2; (**b**) cycle #10; (**c**) cycle #50; (**d**) cycle #200 with $\phi_{avy}=0.4$, k = 6.5, $Q_{1}=4.04\times 10^{-5}$, $Q_{2}=19$, $Q_{3}=0.54\times 10^{-5}$, $Q_{4}=20.9$, *χ* = 3.15, *ζ* = 1, m = 0.5, *Re* = 100, *P* = 10, $G_{0}$ = 0, *κ* = 0.001.

**Figure 10:**
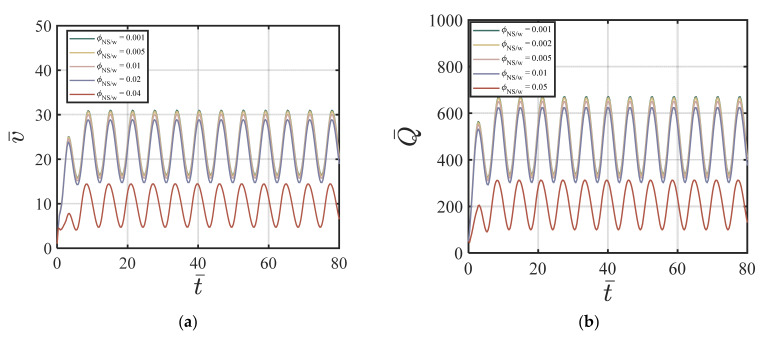
Effect of $\phi_{NS/W}$ of the combined NS FA-added cement suspension on (**a**) $\bar{v}$ vs. *t*; (**b**) $\bar{Q}$ vs. *t* with $\phi_{avy}=0.4$, k = 6.5, $Q_{1}=4.04\times 10^{-5}$, $Q_{2}=19$, $Q_{3}=0.54\times 10^{-5}$, $Q_{4}=20.9$, *χ* = 3.15, *ζ* = 1, m = 0.5, *Re* = 100, *P* = 10, $G_{0}$ = 0, *κ* = 0.001.

Here, we use the following values for our numerical simulations: $\phi_{avy}=0.4$, $Q_{1}=4.04\times 10^{-5}$, $Q_{2}=19$, $Q_{3}=0.54\times 10^{-5}$, $Q_{4}=20.9$, *χ* = 3.15, *ζ* = 1, m = 0.5, *Re* = 100, *P* = 10, $G_{0}$ = 0, *κ* = 0.001. Figure 8a–d show the effects of $\phi_{NS/W}$ on the velocity of the cement suspension with combined nano-silica and fly ash additives at four different time cycles # 2, #10, #50, and #200. From Figure 8a–d, the effect of different values of $\phi_{NS/W}$ on velocity is similar to the NS-added cement suspension case. Larger $\phi_{NS/W}$ values cause smaller centerline velocity and with the increasing time cycle value, the velocity field does not change much. Figure 9a–d show the effects of $\phi_{NS/W}$ on the volume fraction of cement suspension with combined additives at four different time cycles #2, #10, #50, and #200. The volume fraction becomes more uniform in the early time cycle (#10). As shown in Figure 9a–d, after the time cycle 10, the volume fraction profile becomes uniform. Figure 10a,b show the effects of $\phi_{NS/W}$ of the cement suspension with combined additives on the velocity and flow rate versus time. According to Figure 8a–d and Figure 10a,b, the dominance of nano-silica is also exhibited with a similar trend in Section 4.3.1. For the case of combined nano-silica and fly ash particles, we find that the nano-silica particles play a dominant effect, where the velocity and the volume fraction fields show similar trends to the case of only nano-silica-added cement suspension. The finding is validated by Wang et al. [50], that nano-silica particles enhance hydration in early stages and provide favorable hydration conditions for fly ash in later stages. García-Taengua, et al. [20] also showed that fly ash did not affect the dormant period of hydration, while the addition of nano-silica shortened the cement hydrate phase. In addition, the synergistic effect of the two additives increases non-evaporative water content and improves pozzolanic activity. Similar findings are found by Kim, et al. [10], indicating the dominant effect of nano-silica added to high-volume fly ash cement composites, resulting in shorter setting time, higher initial and middle stages of hydration rate, and higher compressive strength.

## 5. Conclusions

In this paper, we look at the flow of a fresh cement suspension in a pipe where two different additives and when they are combined are added to the cement suspension: (1) nano-silica, (2) fly ash, and (3) nano-silica and fly ash. The main findings of the paper are as follows:

1. The cement suspension is modeled as a non-Newtonian fluid, where a modified form of the power-law model is used to study the dependency of the cement suspension viscosity on the shear rate and the volume fraction. The governing equations are made dimensionless, and a limited parametric study is performed for the different dimensionless numbers and parameters.
2. For the parametric study, we focused on the effects of $\phi_{NS/W}$ (the ratio of NS particle volume to the total volume of nano-silica), $\phi_{f}$ (the fly ash content), and the combined two additives; by varying these parameters, we looked at their effects on the velocity and the volume fraction fields.
3. Based on the limited numerical simulations, the results indicate that the velocity and the volume fraction profiles are affected by the addition of different additives. For the pulsating flow of a cement suspension with various additives, we can see that larger values of nano-silica particles can cause a smaller centerline velocity and a higher non-uniformity of the volume fraction field. In contrast to nano-silica particles, larger values of fly ash cause a higher centerline velocity. The effect of fly ash content on the volume fraction is not that obvious.
4. It can also be seen that the fresh cement suspensions with smaller values of nano-silica reach the steady state conditions faster, while the cement suspension with added fly ash particles reaches the steady state very fast.
5. For the case of combined nano-silica and fly ash particles, the nano-silica plays a dominant effect, where the velocity and the volume fraction fields show similar trends to the case of nano-silica-added cement suspension.

These findings can help the design and operation of the pulsating flow of fresh cement mortars and concrete in the 3D printing industry. For future studies, we will do a more systematic parametric study by changing the dimensionless numbers. We will also consider the effect of other factors such as the type of nanoparticles, dispersion of the additives, water-to-cement ratio, particle size and shape, temperature, and the direction of flow on the behavior of fresh cement suspension. In addition, we plan to conduct rheological testing in the laboratory by measuring the viscosity and the yield stress of fresh cement suspensions with various additives under different conditions to check the applicability of the modeling results.

## Figures and Tables

**Table 1:** Values of the dimensionless parameters.

Parameters	Values
$\phi_{NS/W}$	0.001, 0.005, 0.01, 0.02, 0.04
$\phi_{f}$	0.01, 0.05, 0.1, 0.2, 1
$\phi_{avg}$	0.4
k	6.5
χ	3.15
ζ	1
m	0.5
*Re*	100
P	10
$G_{0}$	0
κ	0.001
$Q_{1}$	$4.04\times 10^{-5}$
$Q_{2}$	19
$Q_{3}$	$0.54\times 10^{-5}$
$Q_{4}$	20.9

## Data Availability

Not applicable.

## Nomenclature

**Symbol**	**Explanation**
ρ	Density
g	Acceleration due to gravity
r	Radius of the horizontal pipe
V	Reference velocity
a	Particle radius
$r_{ns}$	Adsorbed water layer thickness for the nanoparticle
$R_{ns}$	Radius of nanoparticles
t	Time
D	Diffusion coefficient
$D_{0}$	Cement suspension diffusivity parameter
$D_{1}$	Diffusion coefficient
$D_{2}$	Volume fraction-dependent diffusion coefficient
η	Constant in volume fraction-dependent diffusion coefficient
$\dot{\gamma }$	Local shear rate
ϕ	Volume fraction of particles
$\phi_{NS/W}$	Volume fraction of nano-silica particles
$\phi_{f}$	Volume fraction of fly ash
$Q_{n}$	Experimental fitting parameters for different types of fly ash
φ	Capillary pore in cement suspension
μ	Viscosity
$\mu_{0}$	Coefficient of viscosity
$\mu_{eff}$	Effective viscosity
$\mu^{*}(\phi )$	Concentration-dependent viscosity
$\mu^{**}(\phi )$	Additive concentration-dependent viscosity
β	Experimental (fitting) parameter for viscosity
$\phi_{m}$	Maximum particle concentration
$\phi_{avg}$	Average volume fraction
p	Pressure
α,m	Material parameters
Re	Reynolds number
$\delta ,G_{0},\kappa ,\zeta ,\chi$	Dimensionless numbers
v	Velocity vector
b	Body force vector
N	Particle transport flux
T	Cauchy stress tensor
$T_{y}$	Yield stress tensor
$T_{v}$	Viscous stress tensor
I	Identity tensor
L	Gradient of the velocity vector
$A_{n}$	n-th order Rivlin–Ericksen tensor
∇	Gradient symbol
div	Divergence operator
